# Progress in flavor research in food: Flavor chemistry in food quality, safety, and sensory properties

**DOI:** 10.1016/j.fochx.2024.102071

**Published:** 2024-12-06

**Authors:** Jeehye Sung, Scott Frost, Joon Hyuk Suh

**Affiliations:** a Department of Food Science and Biotechnology, Andong National University, Andong, 36729, South Korea; b Department of Biology, School of Arts and Sciences, Tufts University, Medford, MA, 02155, USA; c Department of Food Science and Technology, College of Agricultural and Environmental Sciences, University of Georgia, 100 Cedar Street, Athens, GA 30602, USA

## 1 Introduction

Flavor is a critical quality attribute in food and is a primary driver for consumer acceptance and food purchasing decisions. The development of flavor in food is a complex process, involving numerous chemical molecules, including volatiles, non-volatiles and high-molecular weight compounds (Ohloff et al., 1985). The sensation of flavor results from simultaneous stimulation of our chemical senses, mainly odor and taste, triggered by these chemicals originally present in raw food material or generated during food processing and storage. Traditional flavor research focused on identifying key odor-active and taste-active chemicals in food, which are less than a few hundred compounds (Dunkel et al., 2014). Advances in analytical techniques and artificial intelligence (AI), which can handle large datasets, have led recent flavor research toward a more systemic assessment of flavor-related chemicals. This includes the measurement of compounds that are tasteless and odorless but impact flavor perception (e.g., flavor enhancers) and compounds that interact with other molecules to modify flavor profile (Cai et al., 2024; Kuroda, 2024; Ronningen et al., 2018). Studies on flavor precursors and flavor synthetic pathways have also increasingly been introduced in fresh fruits and vegetables, as well as processed food products (Diez-Simon et al., 2019; Xu et al., 2023; Zhu et al., 2019). With these trends, a new omics subdiscipline named ‘flavoromics’ was born in 2008 and has addressed various challenges in food flavor research (Cai et al., 2024). Flavoromics combines analytical chemistry, sensory evaluation, and data science to comprehensively understand the relationships between chemical compositions and flavor traits in food. To characterize a wide range of compounds, including unknown molecules that might affect flavor formation and regulation, flavoromics often employs untargeted chemical analysis using analytical techniques such as gas chromatography–ion mobility spectrometry (GC–IMS), gas chromatography–mass spectrometry (GC–MS) and liquid chromatography–high-resolution mass spectrometry (LC–HRMS) (Castañeda et al., 2024; Wei et al., 2023). A similar term, sensomics, uses a combination of sensory evaluation and instrumental analysis, like flavoromics, while sensomics concentrates on the identification and quantification of aroma-active compounds at the molecular level, which is assisted by experiments such as aroma extract dilution analysis (AEDA) and aroma recombination and omission (Han et al., 2024; Steinhaus & Schieberle, 2007). Multi-omics, the integration of more than one omics approach (e.g., metabolomics with transcriptomics), is another emerging trend to understand in-depth biochemical mechanisms and regulatory processes behind the flavor phenotypes of food across different biological layers (Habibi et al., 2024).

In this special issue, a total of 71 articles regarding the modern flavor chemistry of food were collected between June 2023 and April 2024.

## 2 Articles published in this issue

### 2.1 Flavor chemistry in raw agricultural commodities

Sugars and organic acids are significant chemical components in fresh fruits, contributing to their balanced sweetness and sourness. The total soluble solids to titratable acidity (TSS/TA) ratio is commonly used to assess the flavor quality and ripeness of fruits (Li et al., 2016). However, efforts have been made to measure individual sugars and organic acids for a better understanding of fruit flavor, because each compound may have a different taste activity value (TAV), and their contents and ratios vary among fruits. Sugars, acids, and their proportions and distribution were examined in a cherry species (*Prunus pseudocerasus*) by high-performance liquid chromatography–ultraviolet/refractive index detection (HPLC–UV/RID) (Zhou et al., 2023). With the support of sensory evaluation and the TSS/TA ratio, key indicators of cherry flavor, including glucose, fructose, and maltose were identified, and new cherry grading criteria were proposed. Liu, Song, et al. (2024) explored alterations in flavor-related compounds in yellow and white-fleshed loquats (*Eriobotrya japonica*) using HPLC–UV/RID and LC–MS-based metabolomics approach. Differentially accumulated metabolites, including sugars and organic acids (e.g., malic acid), were found in loquats of different colors and at different development stages.

Interestingly, one collected article used a targeted metabolomics strategy, and reported the identification of ethyl vanillin, a ‘synthetic’ vanilla flavoring compound, in strawberry (*Fragaria* × *ananassa*) (Song et al., 2023). It was the first time that ethyl vanillin had been observed in natural food. The presence of ethyl vanillin in strawberry was thoroughly confirmed by multiple analytical techniques including GC–MS/MS, LC–HRMS and LC–MS/MS with the use of isotope-labeled ethyl vanillin and a global metabolite library (NIST). The same research group published another article regarding the discovery of a novel bitter-masking compound in allspice (*Pimenta dioica*) using sensory-guided isolation (An et al., 2024). The structures of the isolated molecules were determined by nuclear magnetic resonance (NMR) and LC–HRMS, and one molecule exhibited promising bitter masking activity against quinine, as determined by sensory evaluation. Molecular docking analysis indicated that the compound could act as an antagonist of one of the bitter receptors, TAS2R14.

In addition to the above topics, the flavor qualities of buckwheat (*Fagopyrum* spp.) (Li, Wei, et al., 2024), pear (*Pyrus* spp.) (Zhang, Bai, et al., 2024), wampee (*Clausena lansium*) (Zhao et al., 2023), vanilla beans (*Vanilla planifolia*) (Yeh et al., 2024) and pepper seeds (*Capsicum annuum*) (Chen et al., 2024) were assessed by characterizing flavor-related compounds using gas chromatography with flame ionization detection (GC–FID), GC–MS, GC–IMS, HPLC–UV/RID and/or LC–MS/MS, followed by multivariate statistical analysis. One study employed widely targeted metabolomics and annotated nearly 1000 metabolites in pear (Zhang, Bai, et al., 2024). The relationship between metabolites and sensory attributes was uncovered through statistical correlation network analysis, and key sensory-related metabolites in pear were identified based on gene significance and module memberships. Another study examined flavor precursors and downstream aroma volatiles in pepper seeds, demonstrating that certain fatty acids and amino acids were highly correlated with the formation of pepper aroma (Chen et al., 2024).

### 2.2 Flavor chemistry in processed foods

Fifty-five out of the 71 articles in this special issue studied flavor chemistry in processed food products, including marinated and stewed beef (Liu, Deng, et al., 2024; Li et al., 2024), cooked pork (Cheng et al., 2023), sausages (Shao et al., 2024; Sui et al., 2024; Wang, Sui, Liu, et al., 2023), chicken soup (Wang, Wu, et al., 2024), porridge (Liu, Wang, et al., 2024; Wang, Chen, et al., 2024), plant-based meat (Nam et al., 2024), sauerkraut (Wang, Liu, et al., 2024; Wang, Sui, Lu, et al., 2023), coffee (Hong et al., 2024), yogurt (Fan et al., 2024), tea (Li et al., 2023; Liu, Huang, et al., 2024; Long et al., 2024; Ma et al., 2024; Ouyang et al., 2024; Qingyang et al., 2024; Xu et al., 2024; Yan et al., 2024; Zhao et al., 2024), wine (Chen et al., 2023; Gao et al., 2024; Jiang et al., 2024; Qin et al., 2024; Wang, Yin, Shao, et al., 2023; Xi et al., 2024; Zhang, Liu, et al., 2024), soda (Barba et al., 2024), a fruit by-product (Luo et al., 2024), and processed oil (Jeong et al., 2024; Lee et al., 2024; Lin et al., 2024; Zhang, Chen, et al., 2024).

For solid processed foods, key odorants associated with the warmed-over flavor of stewed beef after refrigeration and reheating were characterized using a sensomics approach, which combined sensory evaluation with GC–IMS/MS analysis (Liu, Deng, et al., 2024). Shao et al. (2024) explored the effect of different starter cultures on the microbial communities and flavor compounds of fermented sausages using 16S rRNA gene sequencing and chemical analysis, demonstrating that a mixed starter (*L. plantarum* CQ01107 with *S. simulans* CD207) enhanced the flavor of fermented sausages by generating a high level of umami taste-related compounds. With growing attention on plant-based foods, one study utilized a soybean by-product as an additive for the production of plant-based patties and assessed the quality of the patties via physicochemical measurements and GC–MS analysis (Nam et al., 2024). The developed patties showed increased water-holding capacity, improved texture profiles and lower levels of undesirable flavor volatiles (e.g., benzaldehyde, nonanal, 2-heptanone) compared to control patties without the soybean by-product. Luo et al. (2024) introduced a novel fermented food using a pineapple by-product and whey protein. The effect of fermentation on the product quality was clarified by measuring changes in carbohydrate and amino acid profiles, organoleptic properties, and the microbial community of the product.

For liquid processed foods, the influence of roasting conditions on the physicochemical properties and flavor quality of coffee (*Coffea arabica*) was assessed by analyzing caffeine, chlorogenic acid, total flavonoid content, antioxidant capacity, flavor, and flavor compounds using various instruments such as HPLC–UV, electronic tongue, electronic nose, and GC–MS-olfactometry (GC–MS-O) (Hong et al., 2024). Ouyang et al. (2024) employed untargeted volatile analysis using two-dimensional gas chromatography-olfactometry–quadrupole-time-of-flight mass spectrometry (GC × GC-O–QTOF-MS) to exhaustively characterize the odorants of black tea from different provinces in China. A total of 190 volatiles were identified in the tea, and among them, 23 compounds were confirmed as key odorants contributing to the distinct aromas of black tea in different regions. In another study, the aroma profiles of Italian wines were determined by GC–IMS during spontaneous and inoculated fermentation processes, demonstrating that spontaneous fermentation effectively enhanced the aroma characteristics of wine, with increased levels of corresponding volatiles, showing its aging potential (Wang, Yin, Shao, et al., 2023). Barba et al. (2024) examined the effect of sugar alcohols on the release of aroma compounds in soda beverages using GC–MS and NMR, which may help guide the use of sugar alcohols and aroma volatiles in the formulation of sugar-free or reduced-sugar beverages. Additionally, a new sample preparation technique, called dispersive liquid-liquid microextraction, was introduced and applied to the determination of toxic α-dicarbonyl compounds, such as glyoxal and methylglyoxal, in sesame oil, which has potential for use in the quality and safety control of oil (Lee et al., 2024).

### 2.3 Application of multi-omics in food flavor

Several studies in this issue utilized multi-omics strategies to support and confirm their hypotheses across different biological layers in food products, such as tea (Li et al., 2023; Liu, Huang, et al., 2024), cheese (Xue et al., 2024) and fermented foods (Liang et al., 2023). An integrated metabolomics-transcriptomics approach was employed in oolong tea research, revealing dynamic changes in metabolites and flavor formation during tea manufacturing process (Li et al., 2023). Differentially expressed metabolites and genes were associated, and central metabolic pathways and network (sugar, amino acid and flavonoid metabolism) altered during different tea processing steps were identified. Another study examined variations in metabolite profiles and bacterial communities in Liupao tea during tea processing using a joint metabolomic and metagenomic analysis (Liu, Huang, et al., 2024). The two omics datasets were correlated, and the results indicated that metabolic changes in Liupao tea were not primarily derived from bacterial activities, but rather from other processing conditions, such as high temperature and humidity. A combination of metabolomics and metagenomics was also used in yak cheese (Xue et al., 2024), and fermented dairy and vegetable products (Liang et al., 2023) to elucidate the fundamental mechanisms behind the flavor formation of these products in different contexts.

## 3 Concluding remarks

Chemicals in food are the end products of cellular signaling processes in raw foods or products generated during processing and storage in processed foods. These chemicals directly reflect food phenotypes, such as flavor, which is linked to food quality, safety and sensory properties. This special issue presents recent advances in the flavor chemistry of food and introduces the most up-to-date technologies and approaches for food flavor research. However, challenges remain in the field, as flavor is a complex trait influenced by numerous compounds, their levels, compositions, interactions and reactions. Future advancements in analytical techniques, along with the application of AI technologies such as machine learning and deep learning algorithms, are expected to enhance the measurement and assessment of food flavor from large and complex datasets across various research topics. Finally, the guest editors sincerely thank the authors, reviewers, editors, editorial office, and all those who contributed to this special issue.

## Declaration of competing interest

The authors declare that they have no known competing financial interests or personal relationships that could have appeared to influence the work reported in this paper.
